# Biocompatible carbonized iodine-doped dots for contrast-enhanced CT imaging

**DOI:** 10.1186/s40824-022-00277-3

**Published:** 2022-06-25

**Authors:** Yohan Jeong, Minyoung Jin, Kyoung Sub Kim, Kun Na

**Affiliations:** 1 Department of Biotechnology, The Catholic University of Korea, 43 Jibong-ro, Wonmi-gu, Bucheon-si, Gyeonggi do 14662 Republic of Korea; 2 Department of Research and Developmnet, SML Genetree, Seoul, 06741 Republic of Korea; 3 Department of BioMedical-Chemical Engineering, The Catholic University of Korea, 43 Jibong-ro, Wonmi-gu, Bucheon-si, Gyeonggi do 14662 Republic of Korea

**Keywords:** Iodine-doped dots, CT imaging, Carbon dot, One-pot synthesis, CT contrast agent

## Abstract

**Background:**

Computed tomography (CT) imaging has been widely used for the diagnosis and surveillance of diseases. Although CT is attracting attention due to its reasonable price, short scan time, and excellent diagnostic ability, there are severe drawbacks of conventional CT contrast agents, such as low sensitivity, serious toxicity, and complicated synthesis process. Herein, we describe iodine-doped carbon dots (IDC) for enhancing the abilities of CT contrast agents.

**Method:**

IDC was synthesized by one-pot hydrothermal synthesis for 4 h at 180 ℃ and analysis of its structure and size distribution with UV–Vis, XPS, FT-IR, NMR, TEM, and DLS. Furthermore, the CT values of IDC were calculated and compared with those of conventional CT contrast agents (Iohexol), and the in vitro and in vivo toxicities of IDC were determined to prove their safety.

**Results:**

IDC showed improved CT contrast enhancement compared to iohexol. The biocompatibility of the IDC was verified via cytotoxicity tests, hemolysis assays, chemical analysis, and histological analysis. The osmotic pressure of IDC was lower than that of iohexol, resulting in no dilution-induced contrast decrease in plasma.

**Conclusion:**

Based on these results, the remarkable CT contrast enhancement and biocompatibility of IDC can be used as an effective CT contrast agent for the diagnosis of various diseases compared with conventional CT contrast agents.

**Supplementary Information:**

The online version contains supplementary material available at 10.1186/s40824-022-00277-3.

## Background

Computed tomography (CT) has been used for diagnosing diseases and visualizing fractures based on X-ray attenuation differences in tissues [1]. CT imaging technology has the advantage of being able to inexpensively, quickly, and easily detect and diagnose various diseases with high resolution [2–4]. X-rays are highly absorbed by atoms containing high K-edge energy; however, it is difficult to diagnose diseases of soft tissues except for bone, which is generally rich in iodine and calcium, resulting insufficient contrast intensity for CT imaging [5, 6]. Therefore, studies on various types of iodine-based CT contrast agents have been conducted to aid in the diagnosis of diseases in soft tissues [7, 8]. Intravenously injected mono-molecular iodine moves along with the cardiovascular system, brightens the blood vessels, and is then eliminated through the kidneys and bladder [9]. However, CT contrast agents are known to cause severe pain in patients when injected, and if left for a long time, cause toxicity-related problems such as liver enzymes, lactate dehydrogenase elevations, and normal tissue necrosis [10]. In particular, in the case of CT contrast agents, administration can lead to nephropathy and adverse cardiac events in the clinical fields. For example, patients with iodine-based contrast agent injection showed an increased risk of worsened renal impairment and a significant effect on the development of post-CT acute kidney injury [11–13]. In addition, some CT contrast agents induce adverse cardiac events, such as cardiac death and heart failure, within one month after CT contrast agent administration [14, 15]. To overcome these limitations, studies on contrast agents with high K-edge energy, such as polymers, gold nanoparticles, gadolinium, and bismuth, are being conducted; however, these contrast agents are also not free from low solubility and safety problems [16–21]. Additionally, the synthesis process of these contrast agents relies on complex organic synthesis using hazardous solvents and catalysts [22, 23]. Thus, it is necessary to develop a CT contrast agent that can be prepared by a simple and safe method and has high K-edge energy, high solubility, and minimal toxicity.

In this study, we describe iodine-doped carbon dots (IDCs) that can provide increased CT contrast images and decreased toxicity compared with conventional CT contrast agent, iohexol. Carbon dots (CDs) can be manufactured through a facile one-pot process of simply mixing raw materials and exposing them to high temperatures for a short time without any catalyst or hazardous solvents [24]. CD has the advantages of good biocompatibility and high water solubility, making it suitable as a CT contrast agent [25, 26]. The structural analysis of the synthesized IDC was verified through UV–Vis, XPS, FT-IR, and NMR spectra. The IDC increases the number of iodine atoms per molecule, resulting in a high contrast effect compared to single-molecule iodine, which is iohexol [27]. IDCs showed reduced cytotoxicity at the same concentration of iohexol in human kidney cells. In addition, to confirm the minimized adverse effects of iodine-based CT contrast agents, the toxicity of IDC was evaluated by osmolarity, hemolysis, biochemical, and histological analysis. In particular, the increased CT contrast efficacy of IDC was confirmed with a micro-CT scanner in an animal model. Based on the results of this study, IDC, which shows biocompatible and effective CT images, is expected to be an effective and safe diagnostic nanomaterial with a simple manufacturing process.

## Methods

### Materials

Lactobionic acid (LA), iohexol, iodide standard solution, dimethyl sulfoxide anhydrous (DMSO), and 3-(4,5-dimethyl-2-thiazolyl)-2,5-diphenyl-2H-tetrazolium bromide (MTT) were purchased from Sigma–Aldrich (St. Louis, MO, USA). Phosphate buffered saline (PBS), fetal bovine serum (FBS), Dulbecco’s phosphate buffered saline (DPBS), and 1% antibiotics (streptomycin/penicillin) were purchased from Gibco BRL (Invitrogen Corp., Carlsbad, CA, USA). Dulbecco's modified Eagle’s medium (DMEM) was obtained from HyClone (Losan, UT, USA). DW obtained from a Milli-Q water purification system (Bedford, MA, USA) was used. The dialysis membranes (molecular weight cut off, MWCO: 100–500 Da) were purchased from Spectrum Laboratories Inc. (Rancho Dominguez, CA, USA).

### Synthesis of IDC

Five hundred milligrams of LA and iohexol were dissolved in distilled water (15 mL) and reacted in a 50 mL Teflon-lined hydrothermal chamber to synthesize the IDC. The reaction proceeded at 180 ℃ for 4 h, and the obtained product was cooled at room temperature (RT). The reaction solution was dialyzed against DW for 3 days using a dialysis membrane (MWCO: 100–500 Da) and lyophilized for further use.

### Characterization of IDC

The size distribution and zeta potential of IDC were analyzed using dynamic light scattering (Zetasizer Nano ZS, Malvern Instruments Ltd., UK) at room temperature. The images of the IDC were confirmed with a transmission electron microscope (TEM; JEM-2100, JEOL Ltd, Japan) at 200 kV acceleration voltages. The IDC solutions were dropped onto a copper grid-coated carbon film. To measure the UV–Vis absorption spectra, 0.1 mg/mL of IDC, iohexol, and LA were dissolved in DMSO. Then, UV absorbance was measured with UV–Visible spectrometer (UV-2350, Shimadzu, Japan). X-ray photoelectron spectroscopy (XPS) measurements were conducted using an electron spectroscope (AXIS Supra, Kratos, UK). To analyze the chemical structures of LA, iohexol and IDC, 1H NMR and 13C NMR and Fourier transform infrared (FT-IR) spectrometer (Tensor 27, Bruker, Ettlingen, Germany) were used. 1H NMR and 13C NMR were recorded with 300 MHz NMR spectrometer (Avance III, Bruker, Germany).

### Evaluation of the elemental composition of IDC and iohexol

Elemental analysis of IDC and iohexol were conducted with energy dispersive X-ray spectroscopy (EDX S-10, Oxford instrument, Abingdon, UK) at 15 kV accelerating voltage. Additionally, the iodine contents in the IDC and iohexol were calculated based on the iodide standard solution using inductively coupled plasma atomic emission spectroscopy (ICP–AES, Thermo Fisher Scientific, Bremen, Germany).

### Evaluation of X-ray attenuation properties of IDC and iohexol

X-ray attenuation properties of the IDC were analyzed with HU values of CT images by micro-CT scanner (Quantum GX, Perkin Elmer, MA, USA) at the Daegu Gyeongbuk Institute of Science & Technology (DGIST). The IDC and iohexol solutions were diluted from 0 to 100 mg I/mL with PBS. The HU value of CT images was determined compared with HU values of air (-1000) and water (0).

### Cytotoxicity studies

Madin-Darby Canine Kidney cells (MDCK, KCLB No. 10034) were cultured in Dulbecco's Modified Eagle Medium high (DMEM, HyClone) with 10% heat inactivated fetal bovine serum (FBS) and 1% antibiotics-antimycotics. All cells were cultured in a humidified 5% CO_2_ incubator at 37 °C. The cytotoxicity of IDC and iohexol was evaluated in MDCK cells. MDCK cells (3 × 10^4^ cells per well) were seeded onto 48-well plates and incubated overnight. Various concentrations of IDC and iohexol were added to each well. At 4 h post-treatment, cells were washed twice with DPBS and replaced to complementary medium in each well. After 24 h of incubation, cell viability was evaluated via MTT assay. The absorbance intensity of the solution was measured using microplate reader (Bio-Tek, VT, USA) at 570 nm.

### Biocompatibility test

The osmolality of the IDC and iohexol was measured using Osmomat 030 cryoscopic osmometer (Gonotec, Berlin, Germany) in Korea National University of Transportation, Chungju Center. IDC and iohexol were dissolved in DW at a concentration of 300 mg of iodine. The total osmolality of IDC and iohexol solutions (50 μL) were analyzed in triplicate compared with the freezing point of pure water.

Hemolysis assays were conducted with slight modifications compared with the our previous paper [28]. In brief, fresh mouse blood were centrifuged for 5 min at 3000 rpm to remove the supernatant after collected in EDTA tubes. The blood samples were then washed three times with PBS to obtain mouse red blood cells (MRBCs). The MRBCs were diluted with 0.9% NaCl solution to the 2% (v/v) concentration. The diluted 0.1 mL of MRBC suspension was transferred into 1 mL tubes with 0.9 mL of 0.9% NaCl solution with various concentrations (1–100 mg I/mL) of IDC and iohexol. The mixtures were shaken gently, incubated for 4 h, and centrifuged at 3000 rpm for 10 min. After optical images of the samples were obtained, the absorbance of the supernatant liquids (hemoglobin) was measured using a UV–Vis spectrophotometer. Hemolysis percentages were calculated based on differences in absorbance between the positive (DW) and negative (0.9% NaCl) controls at 540 nm. The degree of hemolysis was calculated as follows:

1 $$\mathrm{Hemolysis pecentage }\left(\mathrm{\%}\right)=\frac{{\mathrm{OD}}_{\mathrm{Positive}}-{\mathrm{OD}}_{\mathrm{Negative}}}{{\mathrm{OD}}_{\mathrm{Positive}}-{\mathrm{OD}}_{\mathrm{Negative}}} X 100$$

### *In vivo* imaging

All procedures were approved by the Institutional Animal Care and Use Committee (IACUC) of the Catholic University of Korea and Daegu Gyeongbuk Institute of Science and Technology in accordance with the “Principles of Laboratory Animal Care,” NIH publication no. 85–23, revised in 1985. Sprague Dawley (SD) male rats (Orient Bio, Inc., Republic of Korea) were used under 22 ± 2 ℃ and 60% relative humidity. 8-week-old male SD rats were fasted for 18 h before CT scans. The CT measurement were set as following conditions: X-ray voltage = 80 kV, anode current = 100 μA, whole-body scan (8 s × 3), field of view (FOV) = 72 mm, and voxel size = 288 μm. All rat was anesthetized before each CT scan. IDC and iohexol (300 mg I/rats) were injected intravenously into SD rats. Each CT image was obtained pre- (before injection), 1 (post-injection), 10, 20, and 30 min after injection.

### *In vivo* toxicity

To evaluate the toxicity of IDC, 30 mg I/mice of iohexol and IDC were injected intravenously into 5-week-old male BALB/c mice. At 24 h after the injection, the major organs (heart, liver, spleen, lung, and kidney) were resected and fixed with 4% paraformaldehyde solution. Major organs were embedded in paraffin, sectioned by a microtome, and stained with hematoxylin and eosin (H&E), and each sample was captured by a slide scanner (Aperio CS2, Leica, Germany). To assess the acute toxicity of IDC, the levels of aspartate aminotransferase (AST), alanine aminotransferase (ALT), blood urea nitrogen (BUN), and creatinine (CRE) were measured after serum was isolated from the blood collected 24 h after the injection.

### Statistical analysis

Data are expressed as the mean ± SD for all the groups. Differences between the values were conducted using one-way ANOVA with Dunnett’s multiple comparison test to the control for PBS and blood plasma. Significance being indicated by *p* values of *P < 0.01 and **P < 0.001. All samples were tested in triplicate.

## Results

### Synthesis and characterization of IDC

**Fig. 1:**
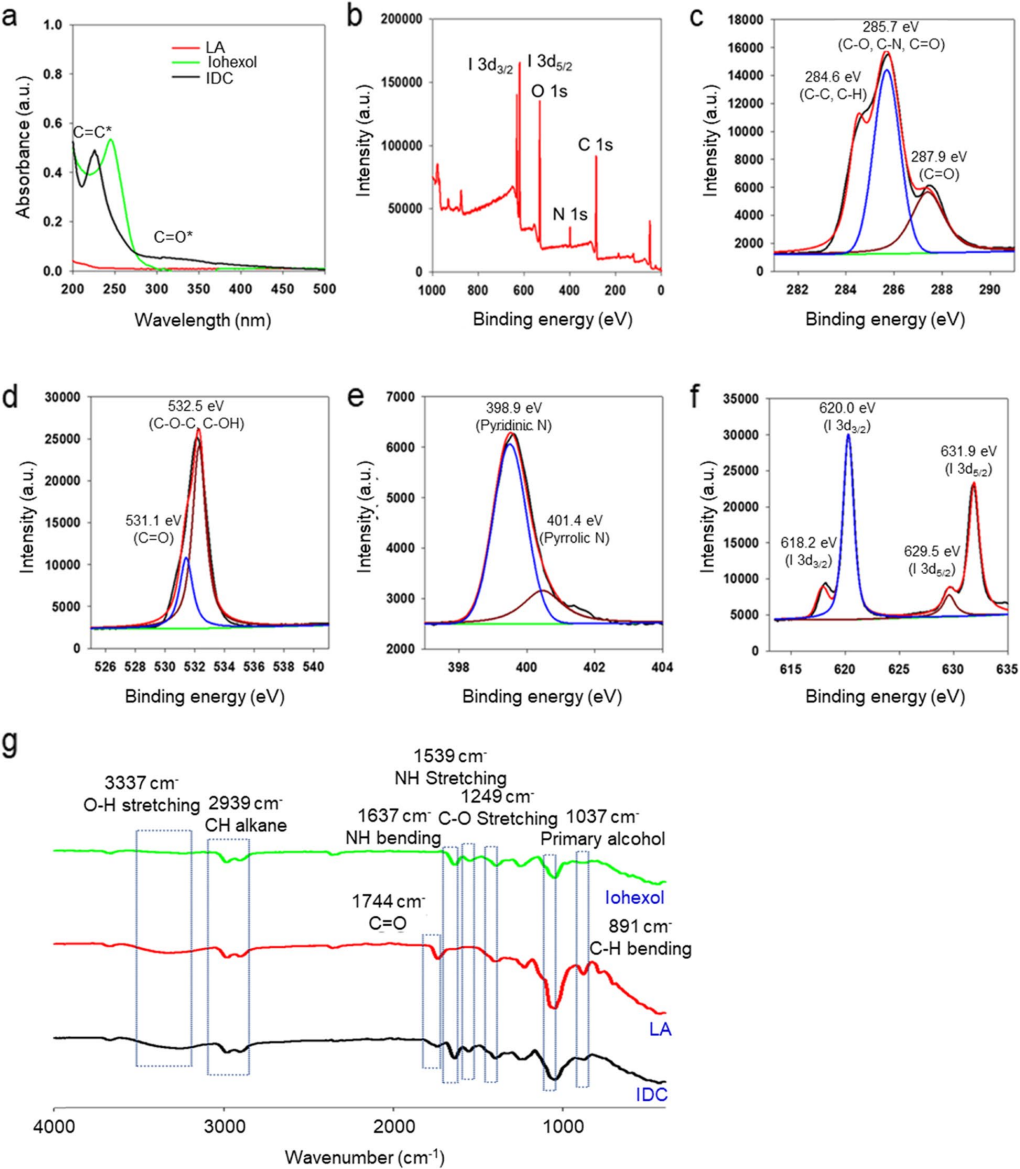
Characterization of IDC **a** UV–Vis spectra of lactobionic acid, Iohexol, and IDC. **b** The full-scan XPS spectrum of IDC. **c** C 1 s, **d** O 1 s, **e** N 1 s, and **f** I 3d XPS spectra of IDC. **g** FTIR spectra of iohexol, lactobionic acid, and IDC

**Fig. 2:**
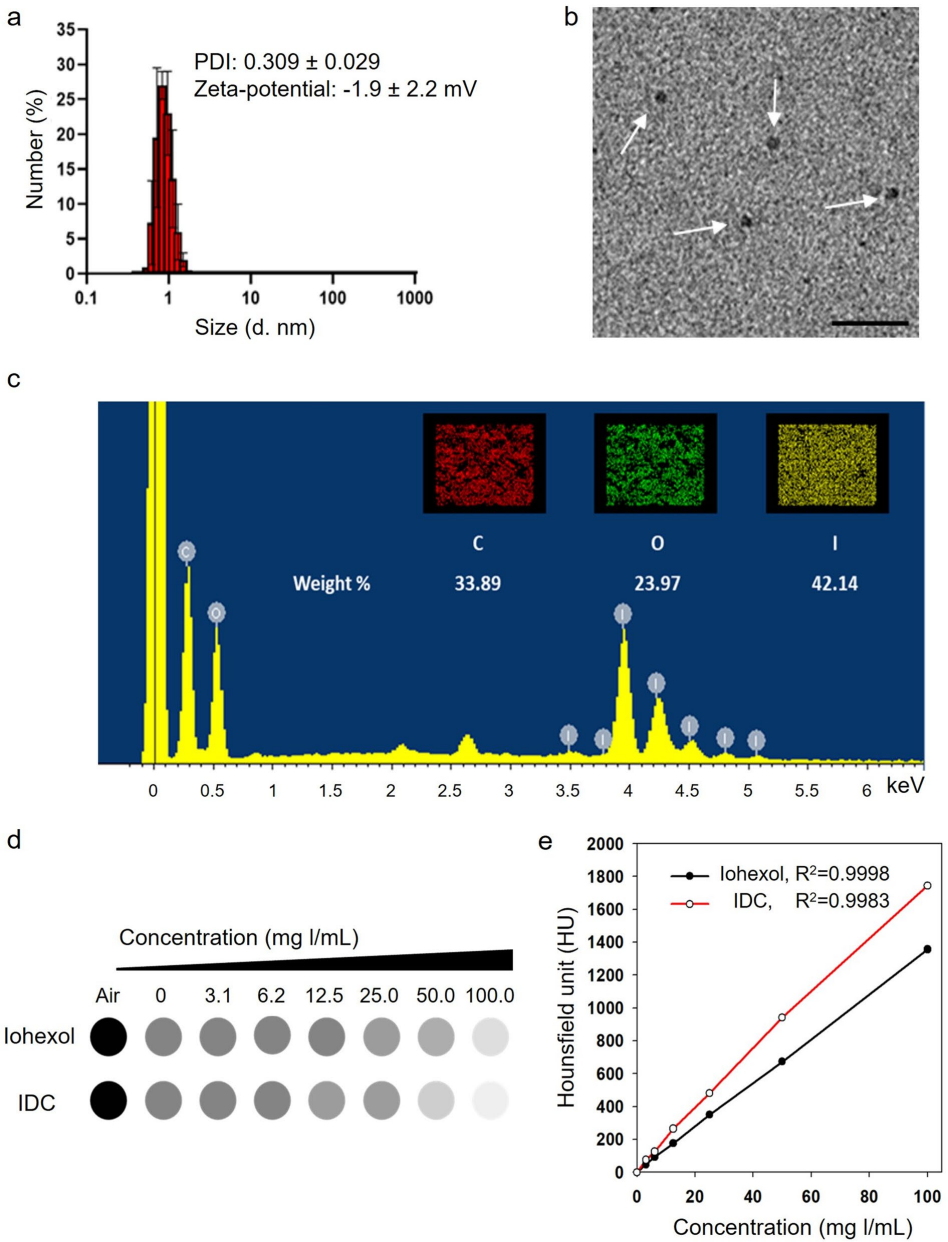
**a** Size distribution of IDC. **b** TEM image of IDC. The white arrow indicates IDC. The black scale bar is 10 nm. **c** EDX element distribution images for C, O, and I in the IDC. **d** In vitro CT imaging of iohexol and IDC. **e** IDC X-ray attenuation linear coefficient with iodine concentration

IDC was synthesized by a one-pot hydrothermal synthesis with LA and iohexol. Both LA and iohexol were dissolved in DW and reacted in a Teflon-lined hydrothermal chamber at 180 ℃ for 4 h. After the reaction, the reactant was purified by dialysis against DW and lyophilized. The synthesized IDC was confirmed by UV–Vis spectra (Fig. 1a). The UV spectrum of IDC showed an iohexol peak shift from 280 to 226 nm and a broad energy peak from 250 to 500 nm. The peak attributed to the aromatic sp^2^ domains (π–π^∗^ transition) was shown at 226 nm, and the peak attributed to the n–π^∗^ excitation was depicted from 250 to 500 nm. To further clarify the chemical structure of IDC, we conducted XPS, FTIR, and NMR analyses. The XPS spectrum of IDC exhibits four main peaks (284.6, 398.9, 532.5, and 620.0 eV) which are assigned to C 1 s, N 1 s, O 1 s, and I 3d, respectively (Fig. 1b). The C 1 s XPS spectrum could be deconvoluted into three peaks at 284.6 eV, 285.7 eV, and 287.9 eV, which contributed to C–C/C-H, C-O/C-N/C = O, and C = O, respectively. (Fig. 1c). The XPS spectrum of O 1 s exhibited two peaks, C = O at 531.1 eV and C–O–C/C–OH at 532.5 eV (Fig. 1d). The XPS spectra of N 1 s showed two peaks for both pyridinic N (398.9 eV) and pyrrolic N (401.4 eV) (Fig. 1e). Two peaks at 620.0 eV and 631.9 eV for 3d_3/2_ and I 3d_5/2_, respectively, are shown in Fig. 1f. In the FTIR spectra, various functional groups of IDC were observed: broad OH peak (3400–3100 cm^−1^), bending of the C = O double bond (1744 cm^−1^), N–H bending (1637 cm^−1^), N–H stretching (1539 cm^−1^), C-O stretching (1249 cm^−1^), primary alcohol (1037 cm^−1^), and C-H bending (891 cm^−1^) (Fig. 1g). ^1^H NMR and ^13^C NMR spectra also simultaneously showed LA and iohexol peaks in IDC (Figures S1 and S2). The IDC has an average size of 4 nm and a weak anionic surface charge of -1.9 mV due to the presence of hydroxyl groups at the surface of IDC (Fig. 2a). The spherical IDC shape and the size of 2–6 nm were confirmed by TEM (Fig. 2b). The iodine content in the IDC was evaluated by SEM–EDX and ICP–AES. Based on EDX analysis, the iodine content in the IDC was determined to be 42.14% (Fig. 2c). ICP–AES analysis data showed that the IDC contained 41.62% iodine, which is consistent with the EDX analysis data.

### X-ray attenuation properties of IDC

To evaluate the X-ray attenuation intensity of the IDC, the CT image and Hounsfield unit (HU) were measured using a micro-CT scanner at various iodine concentrations (0–100 mg l/mL). As shown in Fig. 2d, the IDC exhibited a more prominent positive contrast in the CT image than iohexol with the same iodine concentration. The HU values increased linearly depending on the iodin concentration. IDC has a 28% increased HU value compared to iohexol due to the high iodine atom count per IDC (Fig. 2e).

### *In vitro* toxicity of IDC

**Fig. 3:**
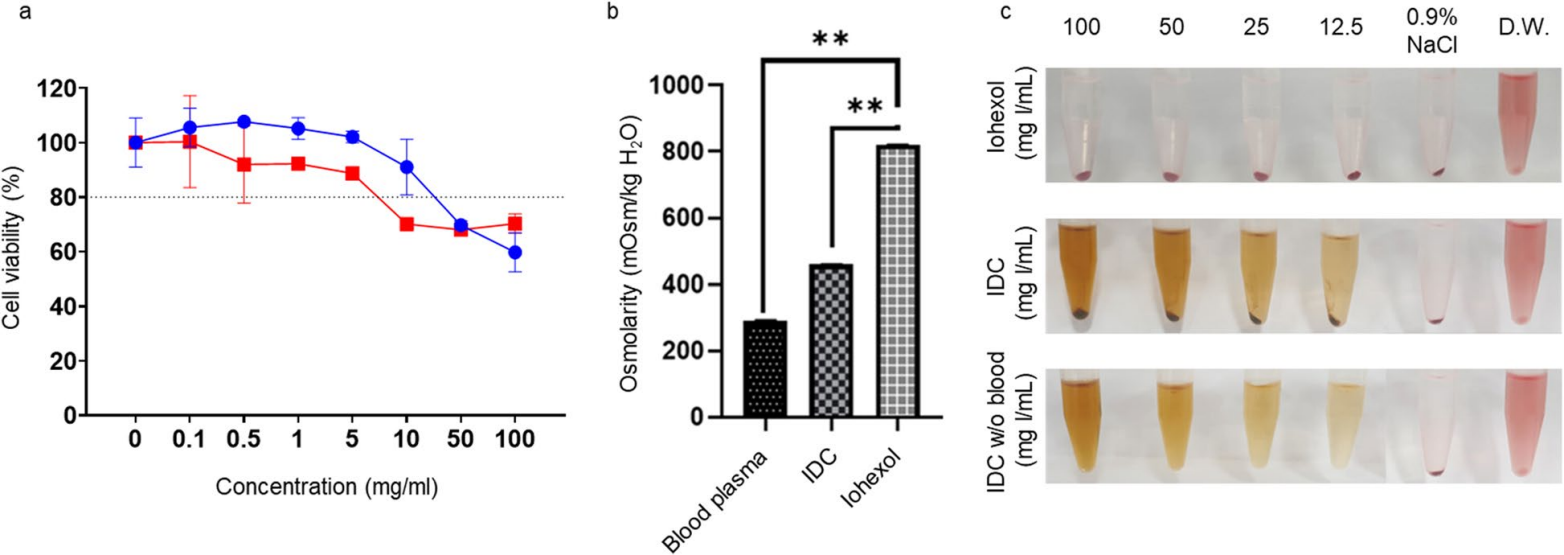
In vitro toxicity of IDC and Iohexol. **a** Cytotoxicity of Iohexol and IDC in MDCK cells. **b** Osmolarity test of IDC and iohexol. **c** Hemolysis assay of iohexol and IDC. Statistical analysis was determined using one‐way ANOVA with Dunnett's multiple comparison test (**P < 0.01)

Since several CT contrast agents can trigger adverse effects in the kidney, liver, and other major organs [29], the cytotoxicity, osmolarity, and hemolysis of IDC were evaluated and compared to those of iohexol. The cytotoxicity of iohexol and IDC was evaluated in MDCK (dog kidney epithelial) cells. Iohexol is cytotoxic at concentrations above 5 mg/mL, whereas IDC is cytotoxic at concentrations above 10 mg/mL. (Fig. 3a). To demonstrate the osmolality of the IDC, the total osmolality of IDC and iohexol solution (300 mg I/mL, 50 μL) was determined in triplicate. The osmotic pressure of iohexol was 2.8 times higher than that of empty plasma, while the osmotic pressure of IDC was 1.5 times higher than that of empty plasma (Fig. 3b). Blood compatibility of IDC was evaluated by a hemolysis assay with the upper limit of the hemolysis index, 5% to clarify the possibility of adverse effects. Even though IDC showed the brown color depending on the IDC’s concentration, hemolytic activity was not observed in either IDC or iohexol at concentrations up to 100 mg I/mL (Fig. 3c).

### *In vivo* CT imaging

**Fig. 4:**
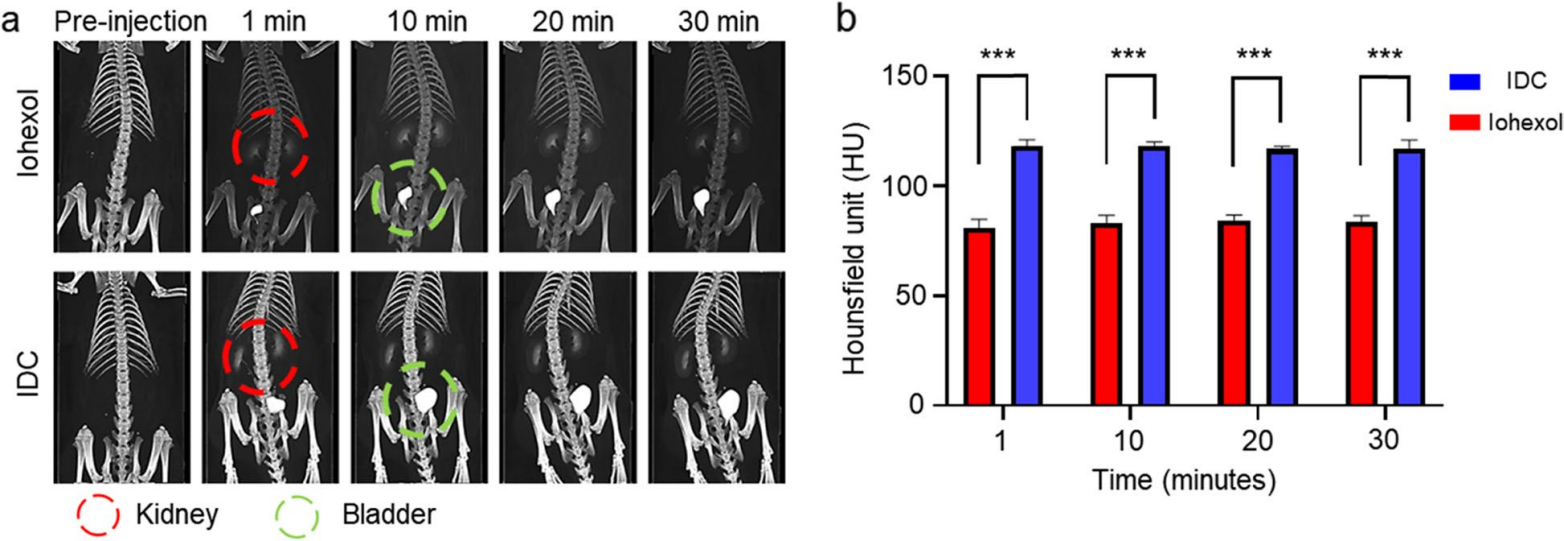
Time-dependent in vivo images **a** Time-course in vivo CT imaging of Iohexol and IDC [X-ray voltage = 80 kV, anode current = 100 Μa, whole-body scan (8 s × 3), FOV = 72 mm, and voxel size = 288 μm]; 200. **b** CT values (HU) in the kidney after intravenous injection of Iohexol and IDC. The statistical analysis was executed via two-way ANOVA with Tukey’s multiple comparison to Pre (***P < 0.001). The data represent the mean ± SD (*n* = 4)

The purpose of synthesizing IDC in this study was to enhance CT contrast enhancement on CT images. IDC and iohexol (300 mg I/rat) were intravenously injected into the tail vein of the SD rats, and CT images were measured over time with a micro-CT scanner. Most contrasting effects were predominantly in the kidney and bladder postinjection of iohexol and IDC (Fig. 4a and Figure S3). The HU value of the kidney in IDC-injected rats increased by approximately 42% compared with the HU value of the kidney in iohexol-injected rats (Fig. 4b).

### *In vivo* toxicity

**Fig. 5:**
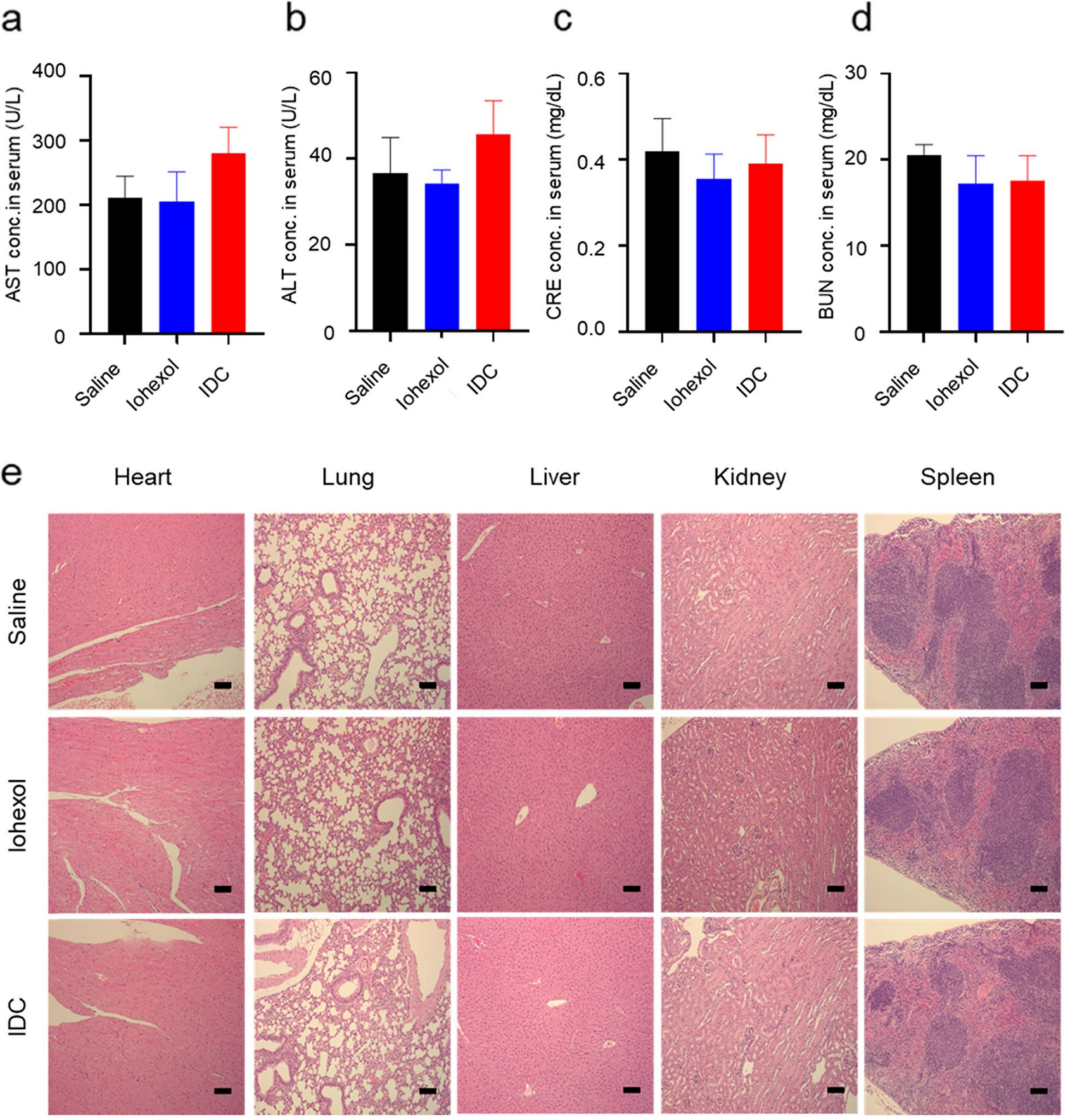
In vivo toxicity in Balb/c mice at 24 h post intravenous injection. **a** Biochemical assay data of mice. Aspartate aminotransferase (AST), alanine aminotransferase (ALT), blood urea nitrogen (BUN), and creatinine (CRE). **b** H&E stained images of harvested major organs including heart, liver, spleen, lung, and kidney from different groups. Black scale bar is 100 μm

To evaluate the in vivo toxicity of IDC, 30 mg I/mice IDC and iohexol were injected intravenously into 5-week-old male Balb/c mice. At 24 h postinjection, blood and major organs (heart, liver, spleen, lung, and kidney) were harvested and analyzed for in vivo toxicity through serum biochemical assays and histological analysis. Serum was isolated from blood obtained from mice treated with iohexol and IDC, and the levels of AST, ALT, CRE, and BUN, which are indicators of renal and hepatic function, were compared with those of the saline-treated group. Negligible differences were measured in liver and kidney health marker levels in the IDC-treated group compared to the saline and iohexol groups (Figs. 5a-d). The harvested major organs were stained by hematoxylin and eosin (H&E). The histological evaluation with H&E staining on major organs showed no obvious pathological changes in major organs (Fig. 5e).

## Discussion

Despite the advantages of relatively inexpensive and fast diagnosis, CT imaging technology has limitations in its application to the diagnosis of diseases of tissues and organs because the K-edge energy is not sufficient for tissues or organs other than the skeleton [5, 6, 30]. Although various types of CT contrast agents are being studied for tissue and organ disease diagnosis through CT imaging technology, high doses of contrast agents need to be administered to obtain sufficient contrast images, which may cause adverse effects [5]. In this study, IDC was synthesized by a one-pot hydrothermal synthesis method without any organic solvent or catalyst to provide improved CT images while minimizing toxicity. IDC was prepared using iohexol and latobionic acid. Iodine-based iohexol (trade name Omnipaque) was selected to manufacture IDC, as one of the most widely used CT contrast agents in clinical practice [31, 32].

IDC confirmed that both the iohexol and LA chemical structures were well maintained and that a new bond (C = O) could also occur through UV–Vis, XPS, FT-IR, and NMR analysis. The synthesized IDC consists of neutral ion spherical nanoparticles with a size of 4 nm containing 42% iodine. IDC provides 28% increased CT contrast enhancement compared to iohexol at the same iodine concentration due to the increased number of iodine atoms per molecule of IDC compared to iohexol. These results indicate that IDC can achieve similar or better CT-contrast effects even at lower doses than iohexol.

IDC exhibited less cytotoxicity than iohexol in canine kidney (MDCK) cells at the same 10 mg/mL iodine concentration. The low osmolality of IDC compared to iohexol can be expected to have a low toxicity probability and high contrast effect since hyperosmotic contrast agents have reduced contrast efficiency due to osmotic dilution [33]. In addition, some conventional contrast agents that have high osmotic pressure cause renal toxicity, pulmonary hypertension, vasodilatation, bradycardia, and even osmotic dilution of contrast [34, 35]. Additionally, IDC exhibits a hemolysis effect that is not significantly different from that of iohexol. These results demonstrate that IDC has a superior CT contrast effect compared to iohexol, as well as reduced cytotoxicity and osmolarity, demonstrating the potential of IDC as a safe and efficient CT contrast agent.

The applicability of IDC as a CT contrast agent was evaluated in the Balb/c mouse model. When IDC was injected intravenously, IDC provided a 42% increase in CT contrast enhancement compared with iohexol in the kidney. According to the principle that foreign substances are eliminated through renal metabolism in the kidney, ICD may accumulate in the kidneys more slowly than iohexol [36]. However, ICD provided a higher contrast enhancement in the kidney than iohexol at the same time postinjection. Although further studies are needed to elucidate the clear circulatory pathway and mechanism of IDC, the increased contrasting effect of ICD in the kidney indicates that the contrast-improving efficacy of IDC can be applied in vitro as well as in vivo.

The biosafety of IDC was proven by biochemical assays and H&E staining images. Since the each enzyme concentration of IDC, saline and iohexol-injected groups all samples were included in the normal ranges [37], IDC could be used for CT contrast agents with superior CT contrast value and biosafety in the field of clinical applications. The histological evaluation with H&E staining on major organs showed no obvious pathological changes in major organs, which indicates their good biocompatibility as a CT contrast agent.

## Conclusions

In this study, an enhanced CT contrast agent was demonstrated via a one-pot hydrothermal reaction with LA and iohexol. IDC was synthesized with a one-pot hydrothermal reaction without any catalyst or organic solvents compared to the complicated synthesis processes of conventional CT contrast agents. UV–Vis spectra of IDC and XPS spectrum showed that IDC is composed of each component and well fabricated as carbon dots. Additionally, the shape of IDC was confirmed as a dot-shaped nanoparticle via TEM images. SEM–EDX and ICP-AES analysis data showed IDC contained high enough iodine contents for CT imaging. In addition, the HU values of the IDC are much higher than conventional CT contrast agent, iohexol, due to the increased iodine atom count per molecule. Furthermore, the HU value of the kidney in IDC injected group was increased compared with the iohexol injected group which is consistent with the in vitro CT imaging. In addition, IDC showed high biocompatibility without blood toxicity, cytotoxicity, or in vivo toxicity. Since IDC provides enhanced CT contrast images without any toxicity, it can be used with a small amount for maximum effects. Thus, IDC will be able to expand the usable range of CT images because IDC has proven its potential for clinical use for safety diagnosis with CT scanners.

## Supplementary Information

**Additional file 1: Figure S1.** ^1^H NMR spectra of LA, iohexol, and IDC. **Figure S2.** ^13^C NMR spectra of IDC in DMSO-d6. **Figure S3.** Time-course in vivo CT 3D imaging of a) iohexol and b) IDC [X-ray voltage = 80 kV, anode current = 100 μA, whole-body scan (8 s × 3), FOV = 72 mm, and voxel size = 288 μm]; 300 mg I/mL.

## Data Availability

Data sharing is not applicable to this article.

## Abbreviations

IDC: Iodine-doped carbon dots
CT: Computed tomography
DW: Deionized water
H: Hours
HU: Hounsfield unit
DMSO: Dimethyl sulfoxide anhydrous
MTT: 3-(4,5-Dimethyl-2-thiazolyl)-2,5-diphenyl-2H-tetrazolium bromide
PBS: Phosphate-buffered saline
DPBS: Dulbecco's phosphate-buffered saline
FBS: Fetal bovine serum
DMEM: Dulbecco's modified Eagle’s medium
FTIR: Fourier transform infrared
EDX: Energy-dispersive X-ray spectroscopy
ICP–AES: Inductively coupled plasma atomic emission spectroscopy
TEM: Transmission electron microscope
OD: Optical density
SD rat: Sprague Dawley rat
MRBC: Mouse red blood cell
FOV: Field of view
AST: Aspartate aminotransferase
BUN: Blood urea nitrogen
ALT: Alanine aminotransferase
CRE: Creatinine
H&E: Hematoxylin and eosin
