# Supplementary material for: Biocompatible carbonized iodine-doped dots for contrast-enhanced CT imaging
Source: Biomater Res. 2022 Jun 25;26:27. doi: 10.1186/s40824-022-00277-3 (PMC9233767; doi:10.1186/s40824-022-00277-3)
Supplement: Supplementary file 1 — Additional file 1: Figure S1. 1H NMR spectra of LA, iohexol, and IDC. Figure S2. 13C NMR spectra of IDC in DMSO-d6. Figure S3. Time-course in vivo CT 3D imaging of a) iohexol and b) IDC [X-ray voltage = 80 kV, anode current = 100 μA, whole-body scan (8 s × 3), FOV = 72 mm, and voxel size = 288 μm]; 300 mg I/mL. [file 40824_2022_277_MOESM1_ESM.docx]

**Supporting information**

**Biocompatible carbonized iodine-doped dots for contrast-enhanced CT imaging**

Yohan Jeong^1,3†^, Minyoung Jin^1,2,†^, Kyoung Sub Kim^1^, Kun Na^1,2,^*

^1^Department of Biotechnology, The Catholic University of Korea, 43 Jibong-ro, Wonmi-gu, Bucheon-si, Gyeonggi do, 14662, Republic of Korea
^2^Department of Biomedical-Chemical Engineering, The Catholic University of Korea, 43 Jibong-ro, Wonmi-gu, Bucheon-si, Gyeonggi do, 14662, Republic of Korea
^3^Department of Research and Developmnet, SML Genetree, Seoul, 06741, Republic of Korea

* Corresponding author: Kun Na, Ph.D.

^†^These authors contributed equally to this work.

Tel.: +82-2-2164-4832

Fax.: +82-2-2164-4865

E-mail: [kna6997@catholic.ac.kr](mailto:kna6997@catholic.ac.kr)


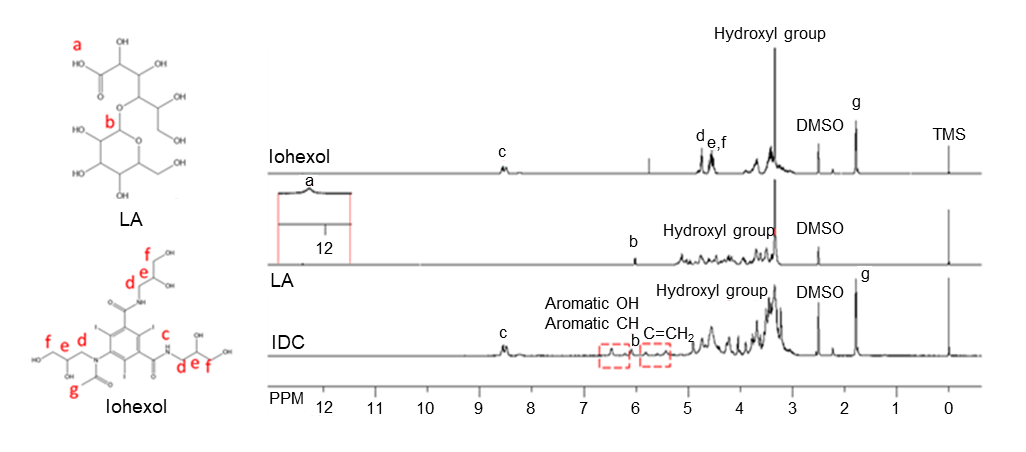


**Figure S1.** ^1^H NMR spectra of LA, iohexol, and IDC.

**
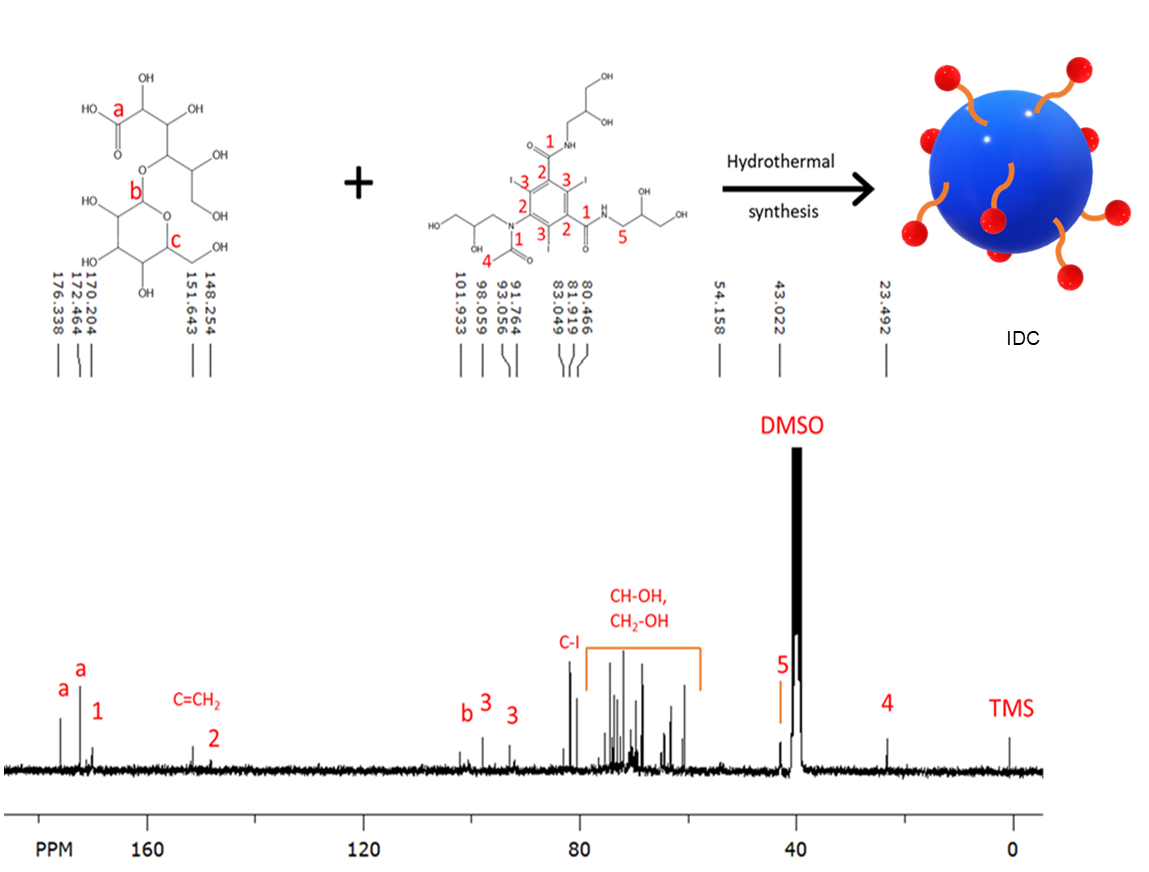
**

**Figure S2.** ^13^C NMR spectra of IDC in DMSO-d6.

**
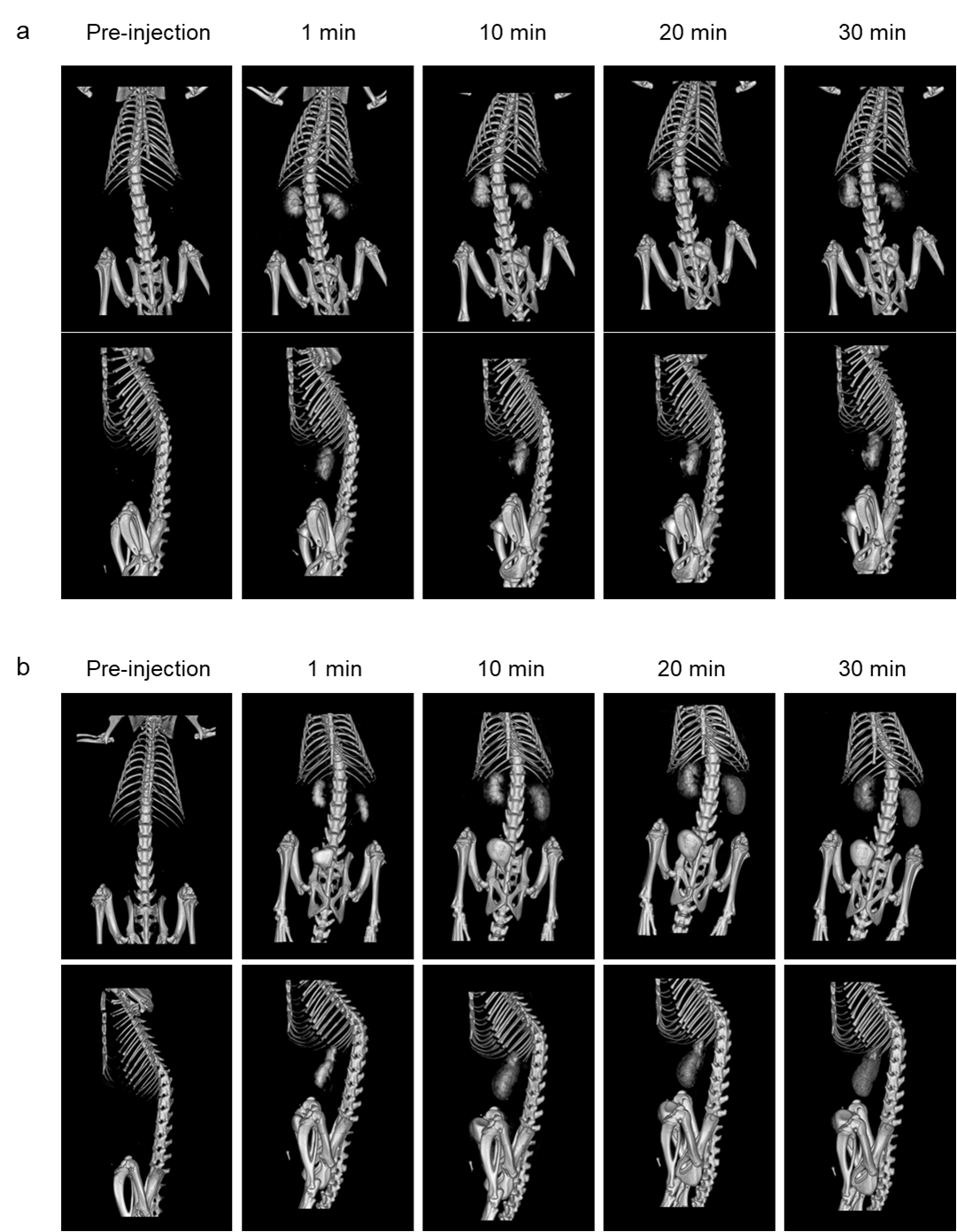
**

**Figure S3.** Time-course in vivo CT 3D imaging of a) iohexol and b) IDC [X-ray voltage = 80 kV, anode current = 100 μA, whole-body scan (8 s × 3), FOV = 72 mm, and voxel size = 288 μm]; 300 mg I/mL.
